# Predicting Adolescents’ Physical Activity Intentions: Testing an Integrated Social Cognition Model

**DOI:** 10.1007/s12529-023-10156-3

**Published:** 2023-03-22

**Authors:** Jessica Balla, Juho Polet, Sami Kokko, Mirja Hirvensalo, Tommi Vasankari, Taru Lintunen, Martin S. Hagger

**Affiliations:** 1 Department of Psychological Sciences, University of California, Merced, USA; 2 Faculty of Sport and Health Sciences, University of Jyväskylä, Seminaarinkatu 15, 40014 Jyväskylän Yliopisto, Finland; 3 Department of Psychology, University of Jyväskylä, Jyväskylän Yliopisto, Finland; 4 UKK Institute for Health Promotion Research, Tampere, Finland; 5 Faculty of Medicine and Health Technology, Tampere University, Tampere, Finland; 6 School of Applied Psychology, Griffith University, Brisbane, Australia; 7 Centre of Excellence in Learning Dynamics and Intervention Research (InterLearn), Faculty of Education and Psychology, University of Jyväskylä, Jyväskylä, Finland

**Keywords:** Theory integration, Exercise behavior, Theory of planned behavior, Habit theory, Health behavior determinants

## Abstract

**Background:**

Few adolescents meet guideline levels of physical activity associated with good health, highlighting the need for intervention. Interventions promoting adolescents’ physical activity should be guided by research applying behavioral theory to identify potentially modifiable correlates and associated processes. We applied an integrated social cognition model to identify theory-based constructs and processes that relate to physical activity intentions in a secondary analysis of two samples of Finnish adolescents using a correlational design.

**Method:**

Participants in the first sample (*n* = 455) completed self-report measures of social cognition constructs from theory of planned behavior, habit, self-discipline, and past and current physical activities. Participants in the second sample (*n* = 3878) completed identical measures plus measures of socio-structural and socio-environmental factors. Participants from the first sample also wore accelerometers for 1 week. Hypothesized model effects were tested using variance-based structural equation modeling in data from the first sample and subsequently confirmed in a pre-registered analysis of data from the second sample.

**Results:**

Across both samples, habit, attitude, perceived behavioral control, and self-reported past behavior were associated with physical activity intention. Effects of self-reported past physical activity on intention were partially mediated by social cognition constructs. Effects of accelerometer-based physical activity were small by comparison. Effects of socio-structural and socio-environmental factors on intention in the second sample were partially mediated by the social cognition constructs.

**Conclusion:**

Results corroborate beliefs and habit as consistent correlates of adolescents’ physical activity intentions and provide preliminary evidence that social cognition constructs account for effects of socio-structural and socio-environmental factors on intentions.

**Supplementary Information:**

The online version contains supplementary material available at 10.1007/s12529-023-10156-3.

## Introduction

Moderate-to-vigorous physical activity participation during childhood and adolescence is associated with multiple health benefits, including reduced chronic disease risk [1] and optimal psychological functioning [2]. Physical activity levels in young people also tend to track into adulthood, offering further protection from chronic disease risk [3]. However, most adolescents worldwide do not meet the World Health Organization [4] daily guideline levels of a daily average of 60 min of moderate-to-vigorous physical activity. Specifically, research suggests that about 81% of adolescents worldwide do not meet these guidelines [4]. Health policy organizations worldwide have, therefore, identified promotion of physical activity in young populations as a priority [5]. Thus, there is a need to develop optimally efficacious behavioral interventions to promote physical activity in young populations. Such interventions should be based on knowledge of the fundamental determinants that drive physical activity participation and the processes involved. To this end, researchers have applied psychological theories to provide an evidence base to inform behavior intervention development. The value of these theories lies in their capacity to identify correlates of physical activity in adolescents that can be potentially modified through intervention.

Theories of social cognition have featured prominently in research seeking to identify these correlates [6]. Such theories focus on psychological constructs that reflect the belief-based considerations in which individuals engage prior to making decisions to act, such as deciding to engage in a health behavior like physical activity [7]. Examples of social cognition beliefs include beliefs about the utility of the behavior in producing desired or useful outcomes, or *attitudes*, and beliefs in personal capacity to perform the behavior in the future, or *perceived control* or *self-efficacy* [8, 9]. However, such theories have been criticized for the assumption that behavior is exclusively a function of a deliberative decision-making process. This has led researchers to incorporate additional constructs that represent other important processes in behavioral performance and to provide a more comprehensive account of the determinants of physical activity. Such approaches are expected to account for a greater proportion of explained variance in physical activity intentions and behavior. These integrated models have incorporated variables that reflect the influence of social structure (e.g., access to resources, socio-economic status) and social environment (e.g., friend and peer support toward physical activity in general) on behavior, and constructs that represent non-conscious processes (e.g., measures of habit or behavioral automaticity) that lead individuals to form intentions and enact behavior through less deliberation. However, the number of research applying these extended theories is relatively few, particularly when examining the determinants of physical activity in adolescents.

To address this evidence gap, the current study sought to identify salient, potentially modifiable correlates of intention to participate in physical activity among Finnish adolescents using an integrated model informed by multiple theoretical perspectives, including theories of social cognition and habit, and models that have incorporated individual difference and socio-structural and socio-environmental factors as additional determinants of intention and behavior. This research is expected to contribute to an evidence base of viable, potentially modifiable constructs that could be the target of interventions to promote physical activity in this population.

## An Integrated Approach to Physical Activity Determinants

Social cognition theories have been frequently applied to identify the determinants of health behaviors, including physical activity [10]. Prominent among these theories is the theory of planned behavior [11]. A key prediction of the theory is that intention toward the future performance of a given target behavior (e.g., physical activity) is the most proximal predictor of that behavior. Intention is a function of three belief-based constructs: attitude, an individual’s positive or negative evaluation with respect to performing the behavior in the future; subjective norm, an individual’s belief that significant others want them to perform the behavior in the future; and perceived behavioral control, an individual’s belief concerning their ability to carry out the behavior in the future and overcome obstacles to its performance. Perceived behavioral control is also specified as a direct predictor of behavior when an individual’s perceptions of control closely match their actual behavioral control. Perceived behavioral control is also expected to moderate the relationships between attitude, subjective norm, and intention [11], although these effects have not been consistently tested. The relationships between attitude, subjective norm, perceived behavioral control, and future behavior are expected to be mediated by intention. The theory has been widely applied to predict behavior in various contexts. Meta-analyses of research have supported the direct and indirect effects proposed in the model across behaviors, including physical activity, and populations, including adolescents [12]. There is also meta-analytic support for the moderating effect of perceived behavioral control on the intention-behavior relationship [13].

Despite support for theory predictions, several limitations of the theory have been noted. While the theory explains substantive variance in intentions and behavior across multiple behaviors, a considerable amount of variance in these constructs remains unexplained [14]. The theory also assumes behaviors are a function of belief-based deliberation, represented by the effects of its constructs on intention and behavior, and does not incorporate constructs that represent non-conscious or *automatic* processes that may lead to intention formation or behavioral enactment [15]. To address these limitations, researchers have suggested integrating additional constructs into the theory that could account for these other processes [16, 17].

Past behavior and habit are candidate additional constructs that have been incorporated into social cognition model tests in health contexts [18]. Inclusion of past behavior as an additional predictor of intention and behavior in theories such as the theory of planned behavior provides a test of its sufficiency; if the theory constructs do not uniquely predict intention and behavior independent of past behavior, then the theory is insufficient as an account of behavior [19]. If relations between past behavior and future behavior are accounted for, or *mediated*, by the social cognition constructs, then the theory provides a sufficient explanation of behavioral consistency, and the indirect effects of past behavior mediated by the social cognition constructs illustrate the extent to which intentions and behavior are informed by past experience [20].

Past behavior has also been used as a proxy measure of habit, considering that repeated performance of a behavior may facilitate habit formation [19]. However, past behavior is not a social cognition construct and, therefore, does not formally capture all characteristics of the habit construct, such as the experienced automaticity of the behavior or the omnipresence of stable contexts or cues that covary with behavioral performance [19]. To resolve this limitation, researchers testing habit effects in social cognition theories have turned to self-reported habit measures that aim to capture key characteristics of habit as construct [19, 21]. Within theory tests, self-reported habit is expected to directly predict behavior, or, at least, in the context of complex behaviors like physical activity, their instigation [19]. Research has also shown that habit is associated with intentions to be physically active (e.g., [22]). This effect may be because individuals who have performed behaviors habitually are likely to express intentions and beliefs about performing these behaviors in the future [19]. In fact, effects of habit on intentions may model the extent to which habits serve as a source of information for individuals when they estimate their beliefs and intentions with respect to performing the behavior in the future. Habits are, therefore, expected to predict intentions to perform physical activity, and reflect an alternative process leading to intention formation.

Researchers seeking to extend the predictive capacity of social cognition theories have also included variables that represent socio-environmental effects on intentions and behavior in health contexts, including physical activity. For example, socio-structural and socio-environmental factors have been identified as important correlates of intention and behavior alongside social cognition constructs, although research examining effects of these constructs within these theories is relatively sparse [23]. These socio-environmental and socio-structural factors have been proposed to predict intentions and behavior in health contexts indirectly through the mediation of specific beliefs about the behavior [24]. Such mediation effects reflect the role that social and physical environmental factors play in informing individuals’ beliefs about performing a behavior in the future. For example, individuals who perceive, or have an actual lack of access to, safe and reliable exercise facilities or spaces may have lower confidence in their ability to be regularly physically active. Thus, perceived behavioral control or self-efficacy could be salient mediators for the relationship between structural barriers toward using exercise spaces and intentions and behavior with respect to being physically active. Research has indicated that socio-structural factors, such as income [25] and perceived access to facilities and local opportunities for physical activity [26], and socio-environmental factors, such as perceived peer support [27], predict intentions and behavior mediated by social cognition constructs such as attitudes [28].

In addition to socio-structural and socio-environmental factors, intra-individual traits have also been identified as prominent determinants of physical activity intentions and behavior. In particular, self-discipline, a generalized tendency to initiate and persevere with tasks despite the presence of distractions or availability of more appealing tasks [29], has been identified as a trait that may inform intention formation and performance of health behaviors such as physical activity (e.g., [30]). This is based on the premise that such traits act as a source of information from which individuals draw when estimating their beliefs and intentions to perform a given health behavior in the future. Such predictions reflect how generalized tendencies serve to bias beliefs and intentions. They are therefore considered distal behavioral determinants and predict behavior mediated by social cognition beliefs (e.g., attitudes, subjective norms) and intentions [31]. This hypothesis has been supported in previous research examining self-discipline as a predictor of intention and behavior in physical activity in the theory of planned behavior (e.g., [31]).

## The Present Study

The importance of regular physical activity participation to physical and mental health in adolescents, and the observed low levels of regular physical activity participation in this population, creates an impetus for identifying potentially modifiable psychological and environmental correlates of physical activity intentions and behavior. The present study aimed to contribute to an evidence base of correlates of adolescents’ physical activity intentions in two large samples of Finnish adolescents using an integrated social cognition approach derived from predictions of the theory of planned behavior, a prototypical social cognition theory, and constructs representing non-conscious processes (past behavior, habit), a key individual difference construct (self-discipline), and socio-structural (perceived access to exercise facilities, cost) and socio-environmental (perceived peer and friend support for physical activity) factors. Data for each sample were collected in 2018 and 2020 as part of the larger Finnish School-Aged Physical Activity (FSPA) and Finnish Late Adolescents Physical Activity (LAPA) studies, which aimed to record nationwide information concerning physical activity and related factors, such as attitudes, in samples of Finnish adolescents [32, 33].

**Fig. 1:**
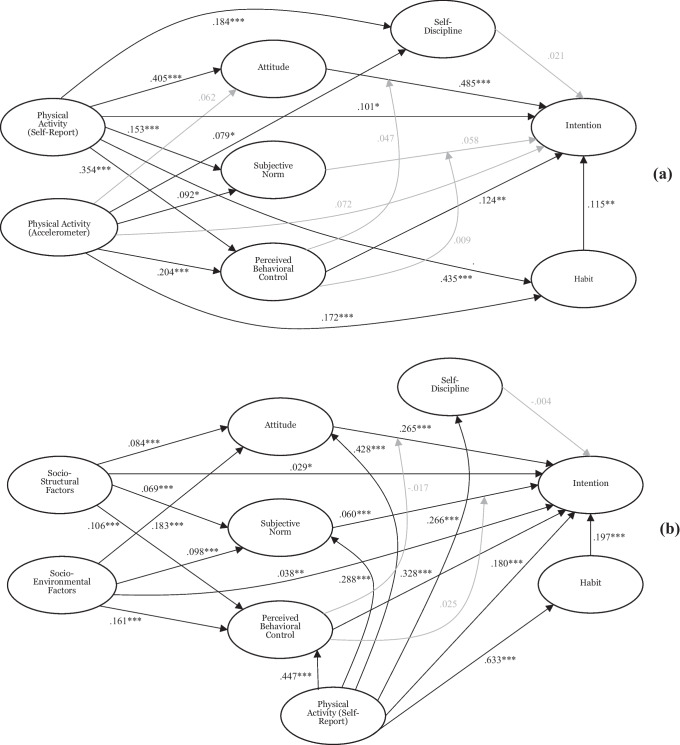
Standardized parameter estimates for the integrated model in the FSPA (**a**) and LAPA (**b**) study samples. Gender, age, residential locale, weight (FSPA sample only), and BMI (LAPA sample only) were included as covariates in the models. *p < .05; **p < .01; ***p < .001

The proposed integrated models, along with the hypothesized relations among the model constructs, are presented in Fig. 1. The first model (Fig. 1a) was tested in the sample from the FSPA study conducted in 2018. We predicted that attitude, subjective norm, and perceived behavioral control would be direct predictors of intention, and that perceived behavioral control would moderate the attitude-intention and subjective norm-intention relationships, consistent with the theory of planned behavior. We predicted that habit and self-discipline would also be direct predictors of intention. We also expected self-reported and accelerometer-based past physical activity behavior to predict intention directly, and also indirectly via the social cognition constructs and habit, consistent with prior research [21, 41].

The second model (Fig. 1b) was tested in the sample from the LAPA study conducted in 2020. In this model, we conducted a pre-registered analysis aimed at replicating key predictions from the model tested in the FSPA study sample and included perceived socio-structural and socio-environmental factors as additional predictors of intention. Specifically, we expected that the pattern of effects of the social cognition constructs and self-discipline specific in the first model would be replicated in the second model. In additional analyses that were not pre-registered, we expected that perceived socio-structural and socio-environmental factors would predict physical activity intentions, and the effects would be mediated by the social cognition constructs in the model, consistent with previous research [8, 49]. Hypotheses relating to habit and theory of planned behavior moderation effects were not pre-registered, but were common across the models.

Our procedure involved testing the hypotheses of the first proposed integrated model in the existing FSPA study sample and, subsequently, pre-registering and testing these hypotheses using data from the LAPA study sample (https://osf.io/h75p4/). The research team pre-registered the proposed model hypotheses prior to receiving the sample data from the LAPA study from the data custodians and performed the analyses once it was received—the research team conducting the pre-registered analyses was not involved in the collection or management of the data. An email trail is available to verify the chain of custody of the data to verify pre-registration which occurred prior to receipt of the data. Tests of hypotheses in the LAPA study sample concerning habit, perceived socio-structural and socio-environmental factors, and theory of planned behavior moderation effects should be considered exploratory.

## Method

### FSPA Study Sample

#### Participants and Recruitment

Participants in the FSPA study sample were children and adolescents aged 7 to 15 years attending Finnish- or Swedish-speaking schools in Finland. Schools (*N* = 311) were recruited using a random selection procedure. Schools were randomly sampled from the Statistics Finland database according to Health Behavior of School-aged Children (HBSC) protocol [52], and students were then randomly selected from the schools that agreed to participate in the study. Students (*N* = 9940) were approached to participate in the study, with 7132 agreeing to complete the final survey. In addition, a subsample of the students (*N* = 3013) consented to wear an accelerometer with useable accelerometer data available from 2782 participants. Written informed consent from both the student and their parent or caregiver was required for participation in accelerometer measurements, while participation in the survey did not require consent; however, parent or caregivers could withdraw their child from the study at their discretion, and information regarding the research was provided to both children and parents. A subsample of participants from the main study (*n* = 455; girls, *n* = 285; boys, *n* = 170; *M* age = 12.65, *SD* = 1.66) that completed the social cognition and psychological measures comprised the final sample used in the current study. This subsample of participants did not have any missing data for the accelerometry measures. Data were collected from March 2018 to May 2018. Full sample characteristics are shown in Appendix B (supplemental materials).

A statistical power analysis was conducted using the inverse square root and gamma-exponential methods for the variance-based structural equation model [53]. Results indicated that to detect a small absolute effect size of 0.250 with a significance level set at 0.05 and a power level of .800, sample sizes of 99 and 86, respectively, were required. An absolute effect size of 0.250 was chosen based on the averaged effect sizes for social cognition constructs on intention found in tests of similar models [54].

#### Design and Procedure

A cross-sectional correlational study design was adopted. Students consenting to participate completed self-report measures of demographic characteristics (age, gender, grade level, locality of residence), social cognition constructs from the theory of planned behavior, self-discipline, habit, and past physical activity. Participants wore an accelerometer for 1 week. Participants completed the questionnaire on a computer or tablet in the classroom under the supervision during a 45-min lesson and a 15-min break. Accelerometers were administered and collected by research assistants or teachers in close proximity to the survey data collection (i.e., a few days before or after the survey data collection) and were worn according to instructions for 7 days. Study procedures were approved by the research ethics committee of the University of Jyväskylä. Full details of data collection methods are reported elsewhere [32, 50].

#### Measures

Study measures comprised validated self-report survey measures alongside an accelerometer measure of physical activity. Full survey measures and response scales are presented in Appendix B (supplemental materials).

##### Demographic Variables

Participants self-reported their demographic characteristics including year of birth, gender, grade level, locality of residence, and mother/father employment status.

##### Social Cognition Constructs

Measures of attitude, subjective norm, and perceived behavioral control were developed according to published guidelines [34]. Attitudes toward physical activity were measured using a common stem (“Participating in active sports and/or vigorous physical activities during my leisure time in the next 5 weeks is…”), with responses measured on two 7-point scales anchored by the bipolar adjectives “unpleasant-pleasant” and “useless-useful.” Subjective norm (“Most people who are important to me think I should do active sports and/or vigorous physical activities during my leisure time for the next 5 weeks”) and perceived behavioral control (“I am confident I could do active sports and/or vigorous physical activities during my leisure time in the next 5 weeks”) were measured using single items with responses provided on 7-point scales (1 = *strongly disagree* to 7 = *strongly agree*).

##### Self-discipline

Self-discipline was measured using six items (e.g., “I start tasks right away”) of the self-discipline scale from the NEO-PI-R [35]. Participants were shown the following instructions prior to completing the measure: “Select the option that describes what kind of person you are usually. Everyone thinks about themselves in a different way so there are no right or wrong answers. Select one option from each row” with responses provided on 5-point scales (1 = *not at all* to 5 = *very much*).

##### Habit

Habit was measured using four items (e.g., “Physical activity is something I do without thinking”) from the Self-Report Habit Index [21]. Responses were provided on seven-point scales (1 = *not true* to 7 = *absolutely true*).

##### Past Physical Activity Behavior

Self-reported past behavior was assessed using two items (e.g., “Think about the last 7 days. On how many days have you exercised at least 60 min a day?”) that captured participants’ frequency of physical activity performed during a usual week. Responses were provided on 8-point scales (0 = *zero days* and 7 = *seven days*).

##### Accelerometer Past Physical Activity

Accelerometer-based physical activity was measured as the average number of minutes spent in moderate (between 3.0 and 5.9 metabolic equivalents, METS) or vigorous (> 6.0 METS) physical activity per day using UKK RM42 accelerometers (UKK Terveyspalvelut Oy, Tampere, Finland). Participants were directed to wear the accelerometers on the hip during waking hours and on the wrist of the non-dominant hand while sleeping. Accelerometers were removed only during aquatic activities. Adequate accelerometer use was defined as wearing the device for at least 4 days out of 7 days, with at least 10 hours of use per day. Accelerometer data were used alongside the past behavior physical activity measures to account for the recall bias associated with self-report methods [36].

## LAPA Study Sample

### Participants and Recruitment

All high schools and vocational schools in Finland (*N* = 371) were invited to participate in the study with 100 schools consenting to participate. A total of 5333 students aged 16 to 20 years consented to participate in the study, with 4958 students from high schools and 375 from vocational schools. A subsample of participants completed the social cognition measures (*n* = 3878; girls, *n* = 2161; boys, *n* = 1694; not reported, *n* = 20; *M* age = 16.64, *SD* = 0.72) and was included in the current analysis. Data were collected using online surveys from September to December 2020. Study protocol was approved by the research ethics committee of the University of Jyväskylä.

### Design and Procedure

The design and procedure of the LAPA study was near identical to that of the FSPA study. However, data for the LAPA study were collected during the COVID-19 pandemic. COVID-19 mitigation policies were enacted in March of 2020, which included restricted access to public facilities, such as sports clubs, and social gatherings comprising more than 10 people; however, measures were taken to enact remote sports instruction in some instances [34]. These restrictions resulted in administration of self-report measures online using Webropol, an online survey tool, rather than in person during collection of data on physical measures. The limitation of group activities may have also limited physical activity participation in the sample overall, so the pattern of effects in the model for this sample should be interpreted accordingly. The online questionnaire had a 60-min time limit to answer all measures. Full details of data collection methods are reported elsewhere [33, 51].

### Measures

The measures administered to participants in the LAPA study sample were the same as those used in the FSPA study, with two notable exceptions. Due to the COVID-19 pandemic, only a small portion of schools took part in the accelerometer measurements; therefore, only self-reported past physical activity behavior was included in the model for this sample. In addition, measures of perceived socio-structural and socio-environmental variables were included for the LAPA study sample, and these measures are described next.

#### Perceived Socio-structural Factors

Perceived socio-structural factors were measured using three items (e.g., “Doing sports/exercise is too expensive”) tapping the perceived social structural elements that may impede physical activity participation, with responses provided on 5-point scales (1 = *not at all* to 5 = *very much*).

#### Perceived Socio-environmental Factors

Perceived socio-environmental factors were measured using two items (e.g., “Appreciation towards exercise among my peers is low”) capturing the perceived social environmental influences expected to affect physical activity participation, with responses provided on 5-point scales (1 = *not at all* to 5 = *very much*).

## Data Analysis

We checked whether the subsamples of participants from the total FSPA and LAPA study samples that responded to the social cognition constructs differed from those who did not complete these measures in terms of gender and age. We also applied Little’s missing completely at random (MCAR) test [37] in each sample with a non-significant value providing evidence that missing cases in each data set were missing completely at random. Analyses were conducted using the SPSS v. 27 software. The hypothesized models illustrated in Fig. 1a, b were tested using data from the FSPA and LAPA study samples, respectively, using variance-based structural equation modeling with the WarpPLS v. 7.0 software. Variance-based structural equation modeling has been recommended for use with data where there is potential for deviation from normality and for estimating complex models [38]. The Stable3 estimation method was used, which provides precise estimates of standard errors [38]. Each construct in the proposed models was a latent variable indicated by its respective items with proposed model relationships included as free parameters. Effects of self-reported past behavior and past physical activity measured via an accelerometer on all social cognition constructs in the model were also included as free parameters. Path coefficient values of 0.02, 0.15, and 0.35 were considered small, medium, and large effect sizes, respectively [38]. Demographic variables such as gender, age, residential locale, and weight or BMI were included as covariates. Residential locale was dichotomized into *urban* and *rural* residents, with urban residents classified as participants who reported living in a city, and rural residents classified as participants who reported living in a village or small town. Missing data were imputed using multiple regression imputation as recommended [38].

Solution estimates were used to evaluate the construct validity, internal consistency, and discriminant validity of the latent variables. Convergent validity was determined by examining the combined factor loadings and cross-loadings after oblique rotation, which should produce statistically significant factor loadings greater than or equal to .500. Internal consistency was assessed using composite reliability coefficients, which should be greater than or equal to .700. Discriminant validity was verified by using the average variance extracted (AVE). The square root of the AVE for all constructs should be greater than the correlations between that variable and other model variables to support discriminant validity.

We used multiple criteria to assess the adequacy of the fit and the quality of the hypothesized models: the Tenenhaus goodness-of-fit (GoF) index, average *R*^2^ (ARS), average full collinearity variance inflation factor (AFVIF), average block VIF (AVIF), average path coefficient (APC), Simpson’s paradox ratio (SPR), *R*^2^ contribution ratio (RSCR), statistical suppression ratio (SSR), and nonlinear bivariate causality direction ratio (NLBCDR). For the Tenenhaus GoF index, an index greater than or equal to .10, .25, and .36, indicates a small, medium, and large effect sizes, respectively. The average *R*^2^, which provides information on a model’s explanatory power, should be statistically significant at the .05 level. The AVIF and AFVIF were used to check for multicollinearity among model variables, and their ideal thresholds are less than or equal to 3.3. The APC, which is based on the absolute values of the path coefficients of the tested model, should have a *p* value equal to or less than .05. The SPR measures the absence of Simpson’s paradox occurrences, which is when a path coefficient has an opposite sign compared to the correlation of the two variables; this implies that the hypothesized path might be reversed in direction or might have issues with causality. The SPR’s ideal threshold should be 1.0, but is acceptable if greater than or equal to 0.7. The RSCR indicates the absence of negative *R*^2^ contributions (when a predictor decreases the amount of variance explained in a criterion variable) and is acceptable if greater than or equal to 0.9, ideally approaching 1.0. The SSR, which measures the absence of statistical suppression with similar implications as Simpson’s paradox, should be greater than or equal to 0.7, ideally approaching 1.0. The NLBCDR provides partial confirmation that the directions of the hypothesized paths are accurate compared to the inverse direction and should ideally be greater than or equal to 0.7.

In addition to sample-specific models estimated in data from the FSPA and LAPA study samples, we also tested for differences in the parameter estimates for the common model effects across samples. This *nested* common model comprised effects of social cognition constructs, self-discipline, and habit on physical activity intentions, moderating effects of perceived behavioral control on the attitude-intention and subjective norm-intention relationships, and effects of self-reported physical activity on all constructs in the model. Effects of accelerometer-based physical activity in the FSPA study sample and effects of perceived socio-structural and socio-environmental variables in the LAPA study sample were not common to both models and not, therefore, subject to the difference tests. Difference tests were conducted using multi-group analysis testing for significant differences in the parameter estimates across the samples using the Satterthwaite method [38].

## Results

### Preliminary Analyses

Preliminary analyses indicated that participants included in the FSPA study sample (*M* age = 12.65, *SD* = 1.66) were significantly older than those who were not (*M* age = 9.61, *SD* = 2.24; *t*(1,911) = 26.71, *p* < .000, *d* = 1.43, CI [2.82, 3.26]). This difference is likely because the social cognition measures were not administered to adolescents in grades 1 and 3, who are typically aged 7 and 11 years, respectively, and were not considered to have sufficient reading ability to comprehend the questionnaires. Participants included in the analysis of the LAPA study sample (*M* age = 16.64, *SD* = 0.72) did not significantly differ in age from those excluded (*M* age = 16.65, *SD* = 0.79; *t*(4,939) =  −.169, *p* = .866, *d* =  −.006, CI [−.05, .04]). There was a larger proportion of girls among participants included in the FSPA study sample (girls, *n* = 285; boys, *n* = 170) relative to those not included (girls, *n* = 811; boys, *n* = 647; *χ*^2^(1, *N* = 1913) = 6.97, *p* = .008, *d* = .120). Similarly, there was a greater proportion of girls among participants included in the LAPA study sample (girls, *n* = 2161; boys, *n* = 1694; not reported, *n* = 20) relative to those that were excluded (girls, *n* = 646; boys, *n* = 413; not reported, *n* = 10; *χ*^2^(2, *N* = 4944) = 10.71, *p* = .005, *d* = .092). Less than 1% of the total data points were missing in both samples. The hypothesis that missing cases were missing completely at random was tested using Little’s MCAR test [36]. The hypothesis was supported in the FSPA study sample (*p* = .540), but not in the LAPA study sample (*p* = .011).

### Structural Equation Models

#### Solution Estimates and Model Fit

Examination of model solution estimates suggested good construct validity for each latent variable, with all factor loadings exceeding .50 with statistically significant coefficients (*p* < .001). Composite reliability estimates for multi-item measures exceeded .700, indicating good internal consistency. Square root of the AVE values for each variable exceeded the correlation between the variable and all other model variables, supporting discriminant validity. Full solution estimates in both samples are presented in Appendix A (supplemental materials). Latent variable correlations for the FSPA and LAPA samples are shown in Appendix D (supplemental materials) and Appendix E (supplemental materials), respectively.

Model fit and quality indices demonstrated adequate fit of the proposed models with the data and acceptable model quality in the FSPA (GoF = 0.468; ARS = 0.239, *p* < .001; AFVIF = 1.898; AVIF = 1.432; APC = 0.115, *p* = .003; SPR = 0.837; SSR = 0.744; NLBCDR = 0.802) and LAPA (GoF = 0.542; ARS = 0.326, *p* < .001; AFVIF = 1.722; AVIF = 1.235; APC = 0.104, *p* < .001; SPR = 0.778; SSR = 1.000; NLBCDR = 0.978) study samples. In addition, the models accounted for a substantial proportion of the variance in physical activity intentions in both samples (FSPA study sample, *R*^2^ = .579; LAPA study sample, *R*^2^ = .727).

#### Model Effects

##### FSPA Study Sample

Standardized path coefficients for the proposed models are presented in Fig. 1, and full parameter estimates and variability and effect size statistics are presented in Table 1. Focusing on the direct effects, we found statistically significant effects of self-reported past physical activity on attitude, subjective norm, perceived behavioral control, self-discipline, habit, and intention. There were also significant effects of past accelerometer-based physical activity on subjective norm, perceived behavioral control, self-discipline, and habit. In addition, there were significant effects of attitude, perceived behavioral control, and habit on intention. Effects of subjective norm and self-discipline on intention, however, were not significant, and perceived behavioral control did not significantly moderate the attitude-intention or subjective norm-intention relationships.

**Table 1:** Parameter and variability estimates for the proposed models in each sample

Effect	*β*^a^	SE	ES
2018 sample			
Direct effects			
PA-SR → SD	.184***	.046	.042
PA-SR → Habit	.435***	.044	.211
PA-SR → Intention	.101*	.046	.045
PA-SR → Attitude	.405***	.045	.166
PA-SR → SN	.153***	.046	.025
PA-SR → PBC	.354***	.045	.144
SD → Intention	.021	.047	.007
Habit → Intention	.115**	.046	.062
Attitude → Intention	.485***	.044	.364
SN → Intention	.058	.047	.025
PBC → Intention	.124**	.046	.081
PA-A → SD	.079*	.046	.015
PA-A → Habit	.172***	.046	.049
PA-A → Intention	.072	.046	.019
PA-A → Attitude	.062	.047	.010
PA-A → SN	.082*	.046	.008
PA-A → PBC	.150***	.046	.052
PBC × Attitude → Intention	.047	.047	.021
PBC × SN → Intention	.009	.047	.003
Indirect effects			
PA-SR → PBC → Intention	.044	.033	.020
PA-SR → Attitude → Intention	.196***	.032	.088
PA-SR → SN → Intention	.009	.033	.004
PA-SR → SD → Intention	.004	.033	.002
PA-SR → Habit → Intention	.050	.033	.022
PA-A → PBC → Intention	.025	.033	.007
PA-A → Attitude → Intention	.030	.033	.008
PA-A → SN → Intention	.005	.033	.001
PA-A → SD → Intention	.002	.033	.000
PA-A → Habit → Intention	.020	.033	.005
Sums of indirect effects			
PA-SR → Intention	.303***	.045	.136
PA-A → Intention	.082*	.046	.021
Total effects			
PA-SR → Intention	.405***	.045	.181
PA-A → Intention	.154**	.046	.040
2020 sample			
Direct effects			
Soc.-Str. → Intention	.029*	.016	.008
Soc.-Str. → Attitude	.084***	.016	.022
Soc.-Str. → SN	.069***	.016	.014
Soc.-Str. → PBC	.106***	.016	.031
Soc.-Env. → Intention	.038**	.016	.012
Soc.-Env. → Attitude	.183***	.016	.059
Soc.-Env. → SN	.098***	.016	.019
Soc.-Env. → PBC	.161***	.016	.050
PA-SR → SD	.266***	.016	.074
PA-SR → Habit	.633***	.016	.402
PA-SR → Intention	.180***	.016	.113
PA-SR → Attitude	.428***	.016	.211
PA-SR → SN	.288***	.016	.097
PA-SR → PBC	.447***	.016	.231
SD → Intention	-.004	.016	.001
Habit → Intention	.197***	.016	.133
Attitude → Intention	.265***	.016	.192
SN → Intention	.060***	.016	.029
PBC → Intention	.328***	.016	.244
PBC × Attitude → Intention	-.017	.016	.008
PBC × SN → Intention	.025	.016	.010
Indirect effects			
PA-SR → PBC → Intention	.147***	.011	.093
PA-SR → Attitude → Intention	.113***	.011	.072
PA-SR → SN → Intention	.017	.011	.011
PA-SR → SD → Intention	-.001	.011	.001
PA-SR → Habit → Intention	.125***	.011	.079
Soc.-Str. → PBC → Intention	.035**	.011	.009
Soc.-Str. → Attitude → Intention	.022*	.011	.006
Soc.-Str. → SN → Intention	.004	.011	.001
Soc.-Env. → PBC → Intention	.053***	.011	.017
Soc.-Env. → Attitude → Intention	.048***	.011	.016
Soc.-Env. → SN → Intention	.006	.011	.002
Sums of indirect effects			
Soc.-Str. → Intention	.061***	.016	.017
Soc.-Env. → Intention	.107***	.016	.035
PA-SR → Intention	.401***	.016	.253
Total effects			
Soc.-Str. → Intention	.090***	.016	.024
Soc.-Env. → Intention	.145***	.016	.047
PA-SR → Intention	.581***	.016	.366

*SE* standard error, *ES* effect size, *SN* subjective norm, *PBC* perceived behavioral control, *PA-SR* moderate-to-vigorous physical activity behavior (self-reported), *PA-A* moderate-to-vigorous physical activity behavior (accelerometer), *SD* self-discipline, *Soc.-Str.* socio-structural factors, *Soc.-Env.* socio-environmental factors

*p < .05; **p < .01; ***p < .001

^a^Standardized path coefficient

Turning to the indirect effects, the effect of self-reported past physical activity on intention through attitude was statistically significant. However, indirect effects of self-reported past physical activity on intention through perceived behavioral control, subjective norm, self-discipline, and habit were not statistically significant. Indirect effects of accelerometer-based past physical activity on intention through all other psychological variables were non-significant.

Sums of indirect effects indicated that the effects of self-reported past physical activity and past physical activity measured via an accelerometer on intention were statistically significant. We also found significant total effects of self-reported and accelerometer-based past physical activity on intention. The significant total effect of accelerometer-based past physical activity was due to the cumulative effect of the small, non-significant effects of accelerometer-based past physical activity on all model constructs which, taken together, translated to a significant total effect.

##### LAPA Study Sample

We found statistically significant direct effects of perceived socio-structural and socio-environmental factors on attitude, subjective norm, perceived behavioral control, and intention. There were also significant direct effects of attitude, subjective norm, perceived behavioral control, and habit on intention, although the effect of self-discipline on intention was not significant. Self-reported past physical activity had significant effects on attitude, subjective norm, perceived behavioral control, self-discipline, habit, and intention.

Indirect effects showed that the effects of self-reported past physical activity on intention through perceived behavioral control, attitude, and habit were statistically significant. In contrast, the effects of self-reported past physical activity on intention through subjective norm and self-discipline were not significant. Indirect effects of perceived socio-structural factors on intention through perceived behavioral control and attitude were significant, while the effect of perceived socio-structural factors on intention through subjective norm was not. The effects of perceived socio-environmental factors on intention through perceived behavioral control and attitude were statistically significant, but the effect of perceived socio-environmental factors on intention through subjective norm was not.

Sums of indirect effects indicated that the effects of self-reported past physical activity, and perceived socio-structural and socio-environmental factors, on intention through all social cognition constructs were statistically significant. In addition, total effects of self-reported past physical activity, and perceived socio-structural and socio-environmental factors, on intention were significant.

### Multi-group Analysis

Testing for differences in parameter estimates common to the models in each sample using multi-group analysis indicated several statistically significant differences: self-reported past physical activity on habit, self-reported past physical activity on subjective norm, self-reported past physical activity on perceived behavioral control, attitude on intention, and perceived behavioral control on intention. In most cases, parameter estimates were larger in the LAPA study sample relative to those in the FSPA study sample. In addition, we found that the moderating effect of perceived behavioral control on the attitude-intention relationship was larger in the FSPA study sample relative to the LAPA study sample. However, while we identified differences in the parameter estimates for these effects across samples, the differences were in the relative size of the effects not in their statistical significance, suggesting that the overall pattern of effects was consistent across samples. Full results from the multi-group analysis are presented in Appendix F (supplementary materials).

## Discussion

We investigated the correlates of physical activity intentions in two samples of adolescents from the FSPA and LAPA studies using an integrated model. The model included social cognition constructs from the theory of planned behavior, which represented reasoned deliberative processes that lead to intention estimation, and constructs and variables representing more non-conscious decision making (past behavior, habit), intra-individual differences (self-control), and perceived socio-structural and socio-environmental factors, all factors likely to be considered when adolescents estimate their physical activity intentions. Structural equation models revealed effects of attitude, subjective norm, perceived behavioral control, habit, self-discipline, and self-reported past physical activity on intention in both samples, with the social cognition constructs mediating the effect of past physical activity on intention. Multi-group analysis revealed a similar pattern of effects across samples, although parameter estimates tended to be larger in the LAPA study sample. We also tested effects of accelerometer-based past physical activity in the FSPA study sample, and effects of perceived socio-structural and socio-environmental factors in the LAPA study sample, on intentions. We observed total indirect effects of perceived socio-structural and socio-environmental factors on intentions mediated by the social cognition constructs, and a total effect of accelerometer-based past physical activity on intention.

### Consistency with Previous Social Cognition Theories

That attitude and perceived behavioral control were consistent predictors of physical activity intentions in both samples is consistent with theory predictions and previous research applying the theory of planned behavior in physical activity (e.g., [39]), although the effect size for the relationship between subjective norm and intentions was smaller and, in the case of the FSPA study sample, not statistically significant. This pattern has been noted in meta-analytic research applying the theory in younger samples and in a physical activity context—attitudes and perceived behavioral control tend to have larger effects on intentions than subjective norms [12]. This is consistent with the notion that beliefs in the utility of physical activity, and in capacity to perform it, are foremost when adolescents make decisions to participate in physical activity. We also found no moderating effects of perceived behavioral control on the attitude-intention and subjective norm-intention relationships, consistent with recent meta-analytic findings [13]. Research seems to more consistently support the moderation of the intention-behavior relationship by perceived behavioral control. However, scale score coverage of the variables included in the interaction may be a possible moderator of these interaction effects, which should be a consideration for future research.

### Value of the Integrated Approach

An important contribution of the current research is that effects of constructs representing non-conscious processes were included alongside the social cognition constructs that typically represent the processes by which individuals make deliberative, reasoned decisions to perform physical activity. This augmentation is consistent with the premise that those forming intentions to perform a behavior in the past are more likely to make similar decisions in the future and, therefore, are more likely to form beliefs and intentions that are align with their past experience. Sure enough, consistent with previous research indicating that past and future behaviors are important sources of information for intention formation [40, 41], current data indicated direct effects of past behavior and habit on intentions. Adolescents, therefore, tend to estimate their intentions toward participating in future behavior by drawing on their past experiences, obviating the need for substantial forethought, or consideration of the current merits and detriments of the upcoming activity, as captured by constructs such as attitudes.

Importantly, the residual effect of past behavior on intention suggests that past behavior does not exclusively reflect habitual intention formation; otherwise, the effect would be entirely subsumed by habit. The residual effect of past behavior on intention may represent effects of other unmeasured variables (e.g., implicit attitudes, identity) or dispositions (e.g., personality) that may bypass more deliberative *routes* to intention formation. By contrast, accelerometer-based physical activity had modest effects on study constructs, but together amounted to a significant total effect. Differences in these patterns of effects may be attributable to recall bias in the self-report measure, or effects of common method variance, both of which have the potential to inflate effects [42]. By contrast, accelerometer-based physical activity is not subject to these kinds of biases. However, there are also limitations with using these types of devices; for example, they do no capture certain types of activity, are subject to interference, and often do not specifically correspond to the target behavior. This does not mean that either measure lacks value, and both likely capture relevant aspects of physical activity, but it is important to recognize their strengths and limitations and indicate the imperative of including self-report and non-self-report behavioral measures of behavior when testing social cognition models.

A dispositional construct that was included as an additional predictor of physical activity intentions in the test of the integrated model in the current study was self-discipline. This construct did not predict intention in either sample, nor did it mediate the relationship between past physical activity and intention. These results are in contrast with previous research that reported associations between self-control and physical activity intentions (e.g., [31]). It may be that self-discipline is less relevant for this specific behavior and population, and there is relatively little research verifying independent effects of this construct on physical activity intentions in younger populations. Other individual difference factors may be worth considering as determinants of physical activity intentions and behavior, such as the activity facet of extraversion from the NEO conceptualization of personality, which has been consistently linked with physical activity intentions and behavior (e.g., [43, 44]).

The indirect effects of perceived socio-structural and socio-environmental factors on physical activity intention mediated by the social cognition constructs identified in the model tested in the LAPA study sample are an important and unique contribution of the current study. These results indicate that participants’ beliefs about the utility, of social norms toward, and personal capacity to perform physical activity are informed by their perceptions of the social and physical environmental barriers or facilitating factors that may hinder or scaffold their physical activity. It is also important to note that residual effects of these factors on physical activity intentions suggest that the mediation effects were partial. The direct effects suggest that the social cognition factors do not fully account for the effects of these factors on intentions, which may reflect the extent that individuals’ perceptions reflect actual barriers or facilitators. However, it may also be the case that the measures of the social cognition constructs are insufficiently precise in capturing individuals’ beliefs with respect to physical activity behavior, or that other unmeasured beliefs may account for the effects of these structural variables, such as anticipated regret, affect, or moral norms. Nevertheless, the indirect effects provide some preliminary evidence of a potential process by which individuals’ social and physical environment relates to intentions, and is consistent with previous theory and research highlighting the importance of beliefs as sources of information that inform individuals’ decisions to act [8, 49].

These results may contribute to the evidence base of correlates of physical activity intentions in young people, which may signal potential intervention targets. These targets include the social cognition constructs, particularly attitudes and perceived behavioral control, and habits given their consistent effects across model tests in the current samples. Such constructs have been shown to be potentially modifiable through behavior change techniques such as persuasive communication [45, 46] and habit formation [46, 47]. But, these results need corroboration as they cannot provide sufficient basis to claim that changing a particular social cognition construct will lead to intention formation, and such effects need to be established through longitudinal or experimental designs that model change. We are, therefore, loath to make recommendations for intervention based on these data alone. Nevertheless, the current data signal theory-related constructs and processes that may serve as targets for future intervention research that could provide corroboration of the direction and causal effects in the model proposed here.

### Strengths, Limitations, and Avenues for Future Research

The current study has several notable strengths. It adopted a robust theoretical approach integrating multiple constructs representing various processes that lead to physical activity intention formation, tested model hypotheses in an initial sample followed by a pre-registered confirmation in a subsequent sample, and used appropriate analyses and robust measures.

Despite these strengths, there are several limitations that restrict the inferences that can be made based on these data. First, the study adopted a correlational, cross-sectional design; thus, causality of the examined relationships cannot be inferred from the data, but rather from the theory alone. Second, the study adopted a single-wave design and did not include a prospective measure of behavior taken on a subsequent occasion, which means we could not account for variance in actual physical activity participation. Further, our study design and lack of follow-up meant we were unable to account for the volitional processes by which intentions are enacted, as proposed in dual-phase models of action (e.g., model of action phases, health action process approach). This precluded inclusion of measures of constructs like action planning or maintenance self-efficacy that might be expected to represent such processes. Third, despite taking a comprehensive approach to understanding physical activity intentions in adolescents by examining non-conscious, socio-structural, and socio-environmental predictors, the present study did not consider other agentic and contextual factors that may have been influential to the formation of physical activity intentions, such as familial and cultural influences, the built environment, or school, local, or national policies on physical activity promotion. Fourth, chronological age is potentially a crude indicator of developmental age, so we could not infer differences in the tested effects due to age across samples derived from the different studies. To permit better inference of developmental implications of differences in the pattern and strength of effects, researchers should consider testing changes in model effects longitudinally in the same sample over time. Fifth, data in the LAPA study sample were not missing completely at random [37]. However, less than 1% of the data points were missing in this sample and the significant test may have been due to the relatively large sample size. Further, the multiple regression imputation method for missing data used did not require data to be missing completely at random [48]. Nevertheless, systematic data *missingness* should be considered a limitation and results should be interpreted accordingly. Finally, data in the LAPA study sample were collected during the COVID-19 pandemic, which may have influenced participants’ responses compared to the FSPA sample, who did not have restrictions on sporting or social activities at the time of data collection.

## Conclusion

The present study identified correlates of physical activity intentions in two samples of Finnish adolescents based on an integrated social cognition approach that incorporated constructs representing non-conscious processes, individual differences in self-control, and perceived structural and socio-environmental factors. Results demonstrated consistent effects of belief-based constructs, self-reported past physical activity, habit, and perceived socio-structural and socio-environmental factors on intention. Perceived socio-structural factors, socio-environmental factors, and self-reported past physical activity were indirectly related to intention via the belief-based constructs. Results highlight the utility of integrating these factors into theories of social cognition to account for the multiple processes that inform intention formation. Findings suggest that utility and capacity beliefs, habit experience, access to exercise facilities and equipment, and past experience are instrumental factors that inform intentions to be physically active in young people. Further research should aim to establish experimental and intervention support for model predictions, measure subsequent behavioral performance over time, and verify model effects in different populations and contexts.

### Supplementary Information

Below is the link to the electronic supplementary material.

Supplementary file1 (DOCX 46 KB)

## Data Availability

The datasets analyzed during the current study are not publicly available as they contain potentially identifying information. Data analysis scripts and output are have been made available online: https://osf.io/h75p4/.
