# Supplementary material for: Predicting Adolescents’ Physical Activity Intentions: Testing an Integrated Social Cognition Model
Source: Int J Behav Med. 2023 Mar 22;31(1):41–54. doi: 10.1007/s12529-023-10156-3 (PMC10032623; doi:10.1007/s12529-023-10156-3)
Supplement: Supplementary file 1 — Supplementary file1 (DOCX 46 KB) [file 12529_2023_10156_MOESM1_ESM.docx]

**Appendix A**

Sample Characteristics and Descriptive Statistics for Study Variables

| Variable | Statistics |
| --- | --- |
| 2018 sample – Finnish School-Aged Physical Activity (FSPA) study |  |
| Participants | 455 |
| Age, M years (SD) | 12.65 (1.66) |
| Gender, n (%) |  |
| Female | 285 (62.6) |
| Male | 170 (37.4) |
| Grade level, n (%)^a^ |  |
| Grade 5 | 203 (44.6) |
| Grade 7 | 127 (27.9) |
| Grade 9 | 125 (27.5) |
| Locality, n (%)^b^ |  |
| City center | 31 (6.8) |
| City, outside center | 249 (55.0) |
| Village center | 85 (18.8) |
| Village, outside center | 88 (19.4) |
|  |  |
| 2020 sample – Finnish Late Adolescents Physical Activity (LAPA) |  |
| Participants | 3,878 |
| Age, M years (SD)^c^ | 16.64 (0.72) |
| Gender, n (%)^d^ |  |
| Female | 2,161 (55.8) |
| Male | 1,694 (43.7) |
| Grade level, n (%)^e^ |  |
| Grade 1 | 1,902 (49.1) |
| Grade 2 | 1,454 (37.5) |
| Grade 3 | 494 (12.8) |
| Other | 23 (0.6) |
| Locality, n (%)^f^ |  |
| City center | 513 (13.3) |
| City, outside center | 1,802 (46.6) |
| Village or town | 1,009 (26.1) |
| Country, rural | 544 (14.1) |

*Note*. ^a^Grades 5, 7, and 9 in Finnish elementary and middle schools; ^b^Two participants did not report their locality; ^c^Five participants did not report their age; ^d^Twenty participants did not report their gender; ^d^Five participants did not report their grade level; ^e^Grades 1, 2, and 3 in Finnish vocational and high schools; ^f^Ten participants did not report their locality.

**Appendix B**

Items and Response Scales for Study Variables

| Variable | Item(s)/measure | Scale/answer options |
| --- | --- | --- |
| 2018 sample |  |  |
| Demographics | Date of birth  Gender  What grade are you in?  What kind of place do you live in now? | Day, month, year  Boy, girl  1^st^, 3^rd^, 5^th^, 7^th^, 9^th^  City center; city, outside center; village center; village, outside center |
| Attitude | Participating in active sports and/or vigorous physical activities during my leisure time in the next 5 weeks is… | 1 = unenjoyable, 7 = enjoyable  1 = useless, 7 = useful |
| Subjective norm | Most people who are important to me think I should do active sports and/or vigorous physical activities during my leisure time for the next 5 weeks | 1 = strongly disagree, 7 = strongly agree |
| Perceived behavioral control | I am confident I could do active sports and/or vigorous physical activities during my leisure time in the next 5 weeks | 1 = strongly disagree, 7 = strongly agree |
| Self-discipline | Select the option that describes what kind of person you are on average. Everyone thinks of themselves differently, so there are no right or wrong answers. Select one option from each row:  I often waste my time  I start tasks right away  I tend to postpone decisions  I like to get to work at once  I need a push to get started  I tend to carry out my plans | 1 = doesn’t describe me at all, 4 = describes me very well |
| Intention | Circle the number that best describes your answer:  I intend to do active sports and/or vigorous physical activities during my leisure time in the next 5 weeks | 1 = strongly disagree, 7 = strongly agree |
| Habit | We would like to know what you think about doing sport and exercise. Choose the best option for each question:  Physical activity is something I do automatically  Physical activity is something I do without having to consciously remember  Physical activity is something I do without thinking  Physical activity is something I start doing before I realize I’m doing it | 1 = not true, 7 = absolutely true |
| Self-reported past behavior | Think about the last 7 days. On how many days have you exercised at least 60 minutes a day? | 0 = on 0 days, 7 = on 7 days |
|  | How much do you exercise during a regular week? | 1 = not at all, 6 = seven hours or more per week |
| 2020 sample |  |  |
| Demographics | Date of birth  Gender  What grade are you in?  What kind of place do you live in now? | Day, month, year  Boy, girl  1^st^, 2^nd^, 3rd  City center; city, outside center; village or town; country, rural |
| Socio-structural variables | To what extent do the following factors prevent you from doing sports and exercise:  In the proximity of my home there is not instruction of a type of sports that I found interesting  Doing sports/exercise is too expensive  In the proximity of my home, there are no appropriate facilities for exercise | 1 = very much, 5 = not at all |
| Socio-environmental variables | To what extent do the following factors prevent you from doing sports and exercise:  My friends do not do sports either  Appreciation for exercise among my peers is low | 1 = very much, 5 = not at all |
| Attitude | Participating in active sports and/or vigorous physical activities during my leisure time in the next 5 weeks is… | 1 = unenjoyable, 7 = enjoyable  1 = useless, 7 = useful |
| Subjective norm | Most people who are important to me think I should do active sports and/or vigorous physical activities during my leisure time for the next 5 weeks | 1 = strongly disagree, 7 = strongly agree |
| Perceived behavioral control | I am confident I could do active sports and/or vigorous physical activities during my leisure time in the next 5 weeks | 1 = strongly disagree, 7 = strongly agree |
| Self-discipline | Select the option that describes what kind of person you are usually. Everyone thinks about themselves in a different way so there are no right or wrong answers. Select one option from each row:  I often waste my time  I start tasks right away  I tend to postpone decisions  I like to get to work at once  I need a push to get started  I tend to carry out my plans | 1 = doesn’t describe me at all, 4 = describes me very well |
| Intention | Circle the number that best describes your answer:  I intend to do active sports and/or vigorous physical activities during my leisure time in the next 5 weeks | 1 = strongly disagree, 7 = strongly agree |
| Habit | We would like to know what you think about doing sport and exercise. Choose the best option for each question:  Physical activity is something I do automatically  Physical activity is something I do without having to consciously remember  Physical activity is something I do without thinking  Physical activity is something I start doing before I realize I’m doing it | 1 = not true, 7 = absolutely true |
| Self-reported past behavior | Think about the last 7 days. On how many days have you exercised at least 60 minutes a day? | 0 = on 0 days, 7 = on 7 days |
|  | How much do you exercise during a regular week? | 1 = not at all, 6 = seven hours or more per week |

**Appendix C**

Factor Loadings, Reliability Estimates, Average Variances Extracted, and Descriptive Statistics for Model Variables

| Construct | FL | CR | AVE | *M* | *SD* | Skew. | Kurt. |
| --- | --- | --- | --- | --- | --- | --- | --- |
| 2018 sample |  |  |  |  |  |  |  |
| Self-reported PA behavior |  | .880 | .786 | 5.991 | 1.073 | -1.017 | 0.254 |
| Item 1 | .887 |  |  |  |  |  |  |
| Item 2 | .887 |  |  |  |  |  |  |
| Self-discipline |  | .811 | .419 | 2.899 | 0.523 | -0.076 | -0.321 |
| Item 1 | .620 |  |  |  |  |  |  |
| Item 2 | .747 |  |  |  |  |  |  |
| Item 3 | .689 |  |  |  |  |  |  |
| Item 4 | .648 |  |  |  |  |  |  |
| Item 5 | .618 |  |  |  |  |  |  |
| Item 6 | .547 |  |  |  |  |  |  |
| Habit |  | .924 | .752 | 5.286 | 1.560 | -0.740 | -0.269 |
| Item 1 | .831 |  |  |  |  |  |  |
| Item 2 | .896 |  |  |  |  |  |  |
| Item 3 | .900 |  |  |  |  |  |  |
| Item 4 | .839 |  |  |  |  |  |  |
| Attitude |  | .929 | .867 | 5.933 | 1.192 | -1.262 | 1.638 |
| Item 1 | .931 |  |  |  |  |  |  |
| Item 2 | .931 |  |  |  |  |  |  |
|  |  |  |  |  |  |  |  |
| 2020 sample |  |  |  |  |  |  |  |
| Socio-structural factors |  | .858 | .671 | 3.972 | 0.986 | -0.805 | -0.170 |
| Item 1 | .877 |  |  |  |  |  |  |
| Item 2 | .711 |  |  |  |  |  |  |
| Item 3 | .859 |  |  |  |  |  |  |
| Socio-environmental factors |  | .905 | .826 | 4.497 | 0.818 | -1.870 | 3.294 |
| Item 1 | .909 |  |  |  |  |  |  |
| Item 2 | .909 |  |  |  |  |  |  |
| Self-reported PA behavior |  | .919 | .851 | 4.448 | 1.696 | -0.021 | -0.686 |
| Item 1 | .922 |  |  |  |  |  |  |
| Item 2 | .922 |  |  |  |  |  |  |
| Self-discipline |  | .856 | .500 | 2.483 | 0.562 | -0.005 | -0.186 |
| Item 1 | .689 |  |  |  |  |  |  |
| Item 2 | .788 |  |  |  |  |  |  |
| Item 3 | .725 |  |  |  |  |  |  |
| Item 4 | .739 |  |  |  |  |  |  |
| Item 5 | .708 |  |  |  |  |  |  |
| Item 6 | .575 |  |  |  |  |  |  |
| Habit |  | .949 | .823 | 4.454 | 1.797 | -0.287 | -0.932 |
| Item 1 | .907 |  |  |  |  |  |  |
| Item 2 | .937 |  |  |  |  |  |  |
| Item 3 | .941 |  |  |  |  |  |  |
| Item 4 | .841 |  |  |  |  |  |  |
| Attitude |  | .905 | .826 | 5.654 | 1.390 | -1.151 | 0.997 |
| Item 1 | .909 |  |  |  |  |  |  |
| Item 2 | .909 |  |  |  |  |  |  |

*Note*. FL = Factor loading of each item on designated factor, coefficients are combined loadings and cross-loadings (oblique-rotated) from partial least squares structural equation model; CR = Composite reliability coefficient from partial least squares structural equation model; AVE = Average variances extracted for factor from partial least squares structural equation model; *M* = Mean; *SD* = Standard deviation; Skew. = Skewness estimate; Kurt. = Kurtosis estimate; PA: Physical activity.

**Appendix D**

| Variable | 1 | 2 | 3 | 4 | 5 | 6 | 7 | 8 | 9 | 10 | 11 | 12 | 13 | 14 |
| --- | --- | --- | --- | --- | --- | --- | --- | --- | --- | --- | --- | --- | --- | --- |
| 1. Sex | — |  |  |  |  |  |  |  |  |  |  |  |  |  |
| 2. Age | .010 | — |  |  |  |  |  |  |  |  |  |  |  |  |
| 3. Weight | -.211^***^ | .650^***^ | — |  |  |  |  |  |  |  |  |  |  |  |
| 4. Locale^a^ | .028 | .321^***^ | .242^***^ | — |  |  |  |  |  |  |  |  |  |  |
| 5. PB | -.080 | -.174^***^ | -.179^***^ | -.122^**^ | — |  |  |  |  |  |  |  |  |  |
| 6. PA | -.224^***^ | -.524^***^ | -.378^***^ | -.212^***^ | .373^***^ | — |  |  |  |  |  |  |  |  |
| 7. SD | .106^*^ | -.185^***^ | -.121^*^ | -.117^*^ | .219^***^ | .163^***^ | — |  |  |  |  |  |  |  |
| 8. Habit | .054 | -.039 | -.086 | -.095^*^ | .482^***^ | .253^***^ | .288^***^ | — |  |  |  |  |  |  |
| 9. Intention | .121^**^ | -.026 | -.044 | -.048 | .446^***^ | .202^***^ | .318^***^ | .534^***^ | — |  |  |  |  |  |
| 10. Attitude | .126^**^ | .005 | -.032 | -.049 | .381^***^ | .144^**^ | .322^***^ | .503^***^ | .748^***^ | — |  |  |  |  |
| 11. SN | .005 | .040 | .044 | -.086 | .157^***^ | .060 | .234^***^ | .282^***^ | .400^***^ | .477^***^ | — |  |  |  |
| 12. PBC | .093^*^ | -.036 | -.067 | -.083 | .394^***^ | .228^***^ | .336^***^ | .508^***^ | .648^***^ | .723^***^ | .385^***^ | — |  |  |
| 13. PBC x Att. | -.116^*^ | .081 | .098^*^ | .020 | -.144^**^ | -.078 | -.070 | -.237^***^ | -.381^***^ | -.553^***^ | -.206^***^ | -.552^***^ | — |  |
| 14. PBC x SN | -.088 | .037 | .093 | .014 | -.055 | -.071 | -.090 | -.148^**^ | -.209^***^ | -.292^***^ | -.092 | -.304^***^ | .492^***^ | — |

Latent Variable Correlations of the Integrated Structural Equation Model Variables for 2018 Sample

*Note*. ^a^Locale was dichotomized as 1 = urban (city residents) and 0 = rural (village or small town residents). PB = Self-reported past behavior; PA = Accelerometer past physical activity; SD = Self-discipline; Hab = Habit; Int = Intention; Att. = Attitudes; SN = Subjective norm; PBC = Perceived behavioral control.

^***^*p* < .001 ^**^*p* < .01 ^*^*p* < .05

**Appendix E**

|  | 1 | 2 | 3 | 4 | 5 | 6 | 7 | 8 | 9 | 10 | 11 | 12 | 13 | 14 | 15 |
| --- | --- | --- | --- | --- | --- | --- | --- | --- | --- | --- | --- | --- | --- | --- | --- |
| 1. Sex | — |  |  |  |  |  |  |  |  |  |  |  |  |  |  |
| 2. Age | .003 | — |  |  |  |  |  |  |  |  |  |  |  |  |  |
| 3. BMI | -.012 | .047^**^ | — |  |  |  |  |  |  |  |  |  |  |  |  |
| 4. Locale^a^ | .007 | -.002 | .005 | — |  |  |  |  |  |  |  |  |  |  |  |
| 5. PB | -.116^***^ | -.118^***^ | .008 | -.041^*^ | — |  |  |  |  |  |  |  |  |  |  |
| 6. SSF | -.112^***^ | -.078^***^ | -.030 | -.203^***^ | .262^***^ | — |  |  |  |  |  |  |  |  |  |
| 7. SEF | .082^***^ | -.037^*^ | -.029 | -.014 | .218^***^ | .422^***^ | — |  |  |  |  |  |  |  |  |
| 8. SD | -.094^***^ | -.088^***^ | -.035^*^ | .027 | .276^***^ | .146^***^ | .127^***^ | — |  |  |  |  |  |  |  |
| 9. Habit | -.101^***^ | -.062^***^ | -.033^*^ | .007 | .629^***^ | .274^***^ | .260^***^ | .331^***^ | — |  |  |  |  |  |  |
| 10. Intention | -.021 | -.014 | -.005 | -.000 | .619^***^ | .270^***^ | .315^***^ | .269^***^ | .669^***^ | — |  |  |  |  |  |
| 11. Attitude | -.011 | -.006 | -.018 | .025 | .480^***^ | .266^***^ | .315^***^ | .270^***^ | .572^***^ | .710^***^ | — |  |  |  |  |
| 12. SN | -.043^**^ | -.055^***^ | .011 | -.023 | .334^***^ | .189^***^ | .184^***^ | .164^***^ | .380^***^ | .480^***^ | .463^***^ | — |  |  |  |
| 13. PBC | -.044^**^ | -.008 | -.017 | -.001 | .509^***^ | .294^***^ | .308^***^ | .224^***^ | .545^***^ | .741^***^ | .665^***^ | .477^***^ | — |  |  |
| 14. PBC x Att. | .023 | -.013 | .026 | -.014 | -.199^***^ | -.116^***^ | -.185^***^ | -.090^***^ | -.269^***^ | -.368^***^ | -.470^***^ | -.228^***^ | -.511^***^ | — |  |
| 15. PBC x SN | -.002 | -.034^*^ | .007 | -.017 | -.097^***^ | -.053^**^ | -.117^***^ | -.027 | -.118^***^ | -.228^***^ | -.263^***^ | -.212^***^ | -.375^***^ | .530 | — |

Latent Variable Correlations of the Integrated Structural Equation Model Variables for 2020 Sample

*Note*. ^a^Locale was dichotomized as 1 = urban (city residents) and 0 = rural (village or small town residents). BMI = Body mass index; PB = Self-reported past behavior; SSF = Socio-structural factors; SEF = Socio-environmental factors; SD = Self-discipline; Att. = Attitude; SN = Subjective norm; PBC = Perceived behavioral control.

^***^*p* < .001 ^**^*p* < .01 ^*^*p* <.05

**Appendix F**

Absolute Parameter Estimate Differences and Variability Statistics from Multi-Group Analysis Comparing 2018 and 2020 Samples

| Effect | β differences | SE |
| --- | --- | --- |
| PB→SD | .056 | .048 |
| PB→Habit | .149^***^ | .047 |
| PB→Intention | .063 | .049 |
| PB→Attitude | .069 | .047 |
| PB→Subjective Norm | .164^***^ | .049 |
| PB→PBC | .105^*^ | .047 |
| SD→Intention | .030 | .049 |
| Habit→Intention | .071 | .049 |
| Attitude→Intention | .179^***^ | .047 |
| SN→Intention | .005 | .049 |
| PBC→Intention | .200^***^ | .049 |
| PBC x Attitude→Intention | .128^**^ | .049 |
| PBC x Subjective Norm→Intention | .006 | .049 |

*Note*. β = Standardized path coefficient; SE = Standard error; PBC = Perceived behavioral control; PB = Past behavior (self-report); SD = Self-discipline.

^***^*p* < .001 ^**^*p* < .01 ^*^*p* < .05
